# Supplementary material for: Barriers facing persons with disability in accessing sexual and reproductive health services in sub-Saharan Africa: A systematic review
Source: PLoS One. 2020 Oct 12;15(10):e0238585. doi: 10.1371/journal.pone.0238585 (PMC7549766; doi:10.1371/journal.pone.0238585)
Supplement: S1 Checklist — (DOC) [file pone.0238585.s001.doc]

| **Section/topic** | **#** | **Checklist item** | **Reported on page #** |
| --- | --- | --- | --- |
| **TITLE** | | |  |
| Title | 1 | **Barriers facing persons with disability in accessing sexual and reproductive health services in Sub-Saharan Africa: a systematic review** | 1 |
| **ABSTRACT** | | |  |
| Structured summary | 2 | **Background:** There is evidence that persons with disabilities often encounter grave barriers when accessing sexual and reproductive health services. To the best of our knowledge, however, no systematic review has been conducted to pull together these pieces of research evidence for us to understand the nature, magnitude and extent of these barriers in different settings in sub-Saharan Africa. We do not yet have a good understanding of the strength/quality of the evidence that exist on the barriers persons with disabilities face when accessing sexual and reproductive health services in sub-Saharan Africa. We therefore conducted a systematic review to examine the barriers persons with disabilities face in accessing sexual and reproductive health services in sub-Saharan Africa.  **Methods:** A systematic review was conducted using PRISMA guidelines (PROSPEROO protocol registration number: CRD42017074843). An electronic search was conducted in Medline, EMBASE, CINAHL, PsycINFO, and Web of Science from 2001 to 2020. Manual search of reference list was also conducted. Studies were included if they reported on barriers persons with disability face in accessing sexual and reproductive health services. The Critical Appraisal Skills Programme and Centre for Evidence Based Management (CEBMa) appraisal tools were used to assess methodological quality of eligible studies.  **Findings**  A total of 1061 studies were identified. Only 26 studies covering 12 sub-Saharan African countries were eligible for analysis. A total of 33 specific barriers including inaccessible physical health infrastructure and stigma and discrimination were identified. These barriers were further categorised into five levels: broader national level barriers; healthcare system/institutional barriers; individual level barriers; community level barriers; and economic barriers.  **Conclusion**  Persons with disabilities face a myriad of demand and supply side barriers to accessing sexual and reproductive healthcare in sub-Saharan Africa. Multilevel interventions are urgently needed to address these barriers | 2 |
| **INTRODUCTION** | | |  |
| Rationale | 3 | Persons with disabilities (PWDs) constitute more than 15% of the world’s population^1^. Disability is the consequence of an impairment that could be physical, cognitive, mental, sensory, emotional, developmental, or some combination of these that result in restrictions on an individual's ability to participate in their everyday society^1^.  PWDs are one of the most marginalised and socially excluded groups in many countries^2–4^. This marginalization transcends several spheres: PWDs have generally poorer health, lower education achievements, fewer economic opportunities and higher rates of poverty than people without disabilities^1^. In particular, women with disability are more likely to be poorer and have lower social and economic status than their counterparts who have no disability^3–5^. In recognition of this, the United Nations Convention on the Rights of Persons with Disabilities guarantees PWDs the fundamental human rights and equitable opportunities to access quality and standard of healthcare^2^. In spite of increased awareness created by the UN Convention, PWDs still face numerous challenges to accessing healthcare^3,5–11^. Impediments to accessing healthcare services include attitudinal biases of health and social service providers, and physical barriers in clinical settings^5–7,9,12,13^.  In the context of sexual and reproductive health, a number of recent studies note that PWDs have been ignored in many low-income settings^14–23^. Part of the reason for this neglect is the impression that PWDs are not sexually active and less likely to marry or have children than persons without disability ^14–23^. Recent evidence however shows that rates of sexual desire and activity, need for family planning services, and childbearing among disabled women are comparable to those of non-disabled women^1,20,21^. In this regard, it is noteworthy that a number of studies within sub-Saharan Africa have started to highlight the challenges PWDs face accessing sexual and reproductive health information and services^7–9,11,14,16,19,20,22,24^. While these studies provide useful evidence on the barriers to accessing sexual and reproductive health services among PWDs in the individual contexts within which they have been conducted, no systematic review has been conducted to pull together these pieces of research evidence for us to understand the nature, magnitude and extent of these barriers across sub-Saharan Africa. Moreover, we do not yet have a good understanding of the strength/quality of the evidence that exist on the barriers persons with disabilities face when accessing sexual and reproductive health services in sub-Saharan Africa. This evidence gap could potentially undermine sub-regional planning and efforts to develop more inclusive sexual and reproductive healthcare policies and programmes that have the potential to propel progress towards the Sustainable Development Goals’ 3 objective of universal and/ or equitable access to skilled and comprehensive sexual, reproductive and maternal health services. | 3 and 4 |
| Objectives | 4 | This systematic review aimed to answer the following inter-related research questions: what is the evidence that PWDs face barriers in accessing sexual and reproductive health (SRH) information and services in sub-Saharan Africa; and what specific barriers do PWDs face in accessing sexual and reproductive health (SRH) information and services in sub-Saharan Africa? | 4 |
| **METHODS** | | |  |
| Protocol and registration | 5 | PROSPEROO protocol registration number: CRD42017074843 | 2 and 4 |
| Eligibility criteria | 6 | Both quantitative and qualitative studies published between 2001 and 2020 were eligible for inclusion in the review. Specifically, studies using such data collection techniques as in-depth interviews, focus groups discussions and surveys that have been conducted at a primary healthcare setting, hospital or community level in sub-Saharan Africa and assessed barriers PWDs face in accessing sexual and reproductive health services were included. Only peer reviewed journal articles were considered. Commentaries, editorials, letters written to editors or policy statements were excluded. The year 2001 was chosen to correspond with the period the UN General Assembly established an Ad Hoc Committee to consider proposals for a comprehensive convention to promote and protect the rights and dignity of persons with disabilities. The work of this Ad Hoc Committee culminated in the adoption of the UN Convention on the Rights of Persons with Disabilities in December 2006, which increased global attention to issues affecting PWDs. | 4 |
| Information sources | 7 | We searched five electronic databases, namely MEDLINE, EMBASE, CINAHL, PsycINFO and Web of Science from 2001 to March 2020 with only English Language restriction. The choice of these databases was based on their indexing coverage of biomedical and allied health journals related to the review topic. | 5 |
| Search | 8 | A Medline search strategy was developed and subsequently adapted and applied to the other databases using the appropriate MeSH or key terms. The reference lists of retrieved studies were screened for additional potentially relevant studies. The search strategy and search terms are reported in **Supplementary File 1.**  **Search No.**   1. (Disability) OR (Disabilities) OR (Impairment) OR (Hearing Impairment) OR (Persons with Disability) OR (Physical and Sensory Impairment) OR (Developmental Disability) OR (Developmental and Intellectual Disability) OR (Deaf) OR (Visual) OR (Blind ) OR (Intellectual Disability) OR (Psychosocial Disability) OR (Albinism) OR (Physical Disability) OR (Mental Disability) 2. (Reproductive Health) OR (Sexual Health) OR (Sexual Education) OR (Sexuality Education) OR (Sexual Health Information) OR (Family Planning Services) OR (Antenatal Care) OR (In-facility Delivery) OR (Antenatal Care) OR (Postnatal Care) OR (Modern Contraception) OR (Abortion) OR (Contraceptives) OR (Condom) OR (Pregnant Women) OR (Health Services) OR (Emergency Obstetric Services) OR (Obstetric Complications) OR (Maternal Health) or (Child Health) OR (Skilled Birth Attendance) OR (Sexuality) OR (Adolescent) OR (Sexuality and Disability) OR (Motherhood) OR (Reproductive Health Services) OR (Birth Control) OR (Pills) OR (Intrauterine Device) OR (IUD) OR (Emergency Contraception) OR (Sterilization) OR (Spermicide) OR (Diaphragm) OR ( Cervical Cap) OR (Contraceptive Implant) 3. (Barriers) OR (Challenges) OR (Experiences) OR (Impediments) OR (Access) OR (Inhibiters) OR (Uptake) OR (Utilization) OR (Compliance) OR (Adherence) 4. (Developing country) OR (Low income country) OR (Low-middle income country) OR (Middle income country) OR (Africa) OR (Africa South of the Sahara) OR (Sub-Saharan Africa ) OR (Central Africa) OR (Southern Africa) OR (Eastern Africa ) OR (Western Sahara) OR (East Africa ) OR (Central African Republic ) OR (West Africa ) OR (Cameroon) OR (Chad) OR (Congo) OR (Democratic Republic of Congo) OR (Congo, Demographic Republic) OR (Congo, Republic) OR (Equatorial Guinea) OR (Gabon) OR (Burundi) OR (Djibouti) OR (Eritrea ) OR (Ethiopia ) OR (Kenya) OR (Rwanda) OR (Somalia) OR (Sudan) OR (Tanzania) OR (Uganda) OR (Angola) OR (Botswana) OR (Lesotho) OR (Malawi) OR (Mozambique) OR (Namibia) OR (Swaziland) OR (Zambia) OR (Zimbabwe) OR (Benin) OR (Burkina Faso) OR (Cape Verde) OR (Cote D'ivoire) OR (Gambia) OR (Gambia, The) OR (Ghana) OR (Guinea) OR (Guinea-Bissau) OR (Liberia) OR (Mali) OR (Mauritania) OR (Niger) OR (Nigeria) OR (Senegal) OR (Sierra Leone) OR (Togo) OR (South Sudan) OR (Madagascar) OR (Comoros) OR (Mauritius) OR (Sao Tome and Principe) OR (Seychelles) OR (South Africa) 5. 1 OR 2 OR 3 6. 4 AND 5   **Limiters:**  2001-2020  Full Text  English Language  Academic/Peer reviewed Journals  Humans | 5 and 27 |
| Study selection | 9 | Two authors searched the results from the five debases. Articles were exported to Endnote reference manager where duplicates were removed. The selection process was systematically conducted and displayed in flow chart in line with the PRISMA guidelines (see **Figure 1**). First, the titles and abstracts of studies were screened using pretested study selection form developed from the inclusion criteria to identify potentially eligible studies. Second, all potentially relevant studies’ titles and abstracts were identified by two authors . The remaining authors independently sampled at least five of the eligible studies following title and abstract screening. To minimize bias, authors did not review prospective studies where they were authors. Full text screening was further conducted by two authors and where disagreement arose about the potential eligibility of a particular article, a third reviewer was involved. Where eligible studies reported insufficient information to support the review process, the corresponding authors of those articles were contacted by one reviewer. All studies which did not meet the eligibility criteria were excluded with the reasons for exclusion provided. Where some eligible studies had missing data or presented insufficient published data, one reviewer contacted the study’s corresponding authors to clarify the missing data and retrieve same where the data was able. | 5 and 6 |
| Data collection process | 10 | In order to ensure consistency and transparency, the data extraction process was facilitated by a standardized evidence table (see **Table 1**) where data on the study’s author (s), setting, aim, study design, methods, population characteristics and key findings were extracted. This was done by two reviewers and where a discrepancy arose, the other two reviewers were invited to resolve the issue before the extraction process proceeded. | 6 |
| Data items | 11 | *Types of population*  Persons with disability in this study included those with physical and sensory impairments, developmental and intellectual disability and psychosocial disability. For inclusion, studies must have been conducted in any country in sub-Saharan Africa, and involve either male or female PWDs who are aged 15years and above. While both age of menarche among girls and sexual debut among boys and girls have declined in recent years, most international policy and research on sexual and reproductive health often focus on age 15 onwards as the starting point of sexual activity and reproduction^11,16,17^. Our focus on 15years and above was therefore informed by this international policy and research literature. Studies which reported the views of healthcare personnel who provide direct sexual and reproductive healthcare services to PWDs as well as community and family members of PWDs were also eligible for inclusion.  *Types of intervention*  Studies which sought to identify barriers PWDs face in accessing sexual and reproductive health services were included. Specifically, PWDs should have accessed or likely to access one of the following: sexual health education and information, family planning, contraception, abortion, antennal care (ANC), health facility childbirth, and postnatal care (PNC) services. | 4 and 5 |
| Risk of bias in individual studies | 12 | To minimize bias, authors did not review prospective studies where they were authors. Full text screening was further conducted by two authors and where disagreement arose about the potential eligibility of a particular article, a third reviewer was involved. Where eligible studies reported insufficient information to support the review process, the corresponding authors of those articles were contacted by one reviewer. All studies which did not meet the eligibility criteria were excluded with the reasons for exclusion provided. Where some eligible studies had missing data or presented insufficient published data, one reviewer contacted the study’s corresponding authors to clarify the missing data and retrieve same where the data was able. | 6 |
| Summary measures | 13 | The outcomes of interest in this review included perceived and actual barriers or challenges PWDs face in accessing sexual and reproductive health services. Such barriers should relate to access to or use of sexual health education and information, family planning, contraception, abortion, ANC, health facility childbirth, and PNC services. | 5 |
| Synthesis of results | 14 | A qualitative synthesis approach was used. Findings were presented narratively and in tables. To enhance reporting transparency, the framework for data synthesis by Popay et al^30^ was used. Here, data were reported using tables, highlighting key and unique barriers to accessing sexual and reproductive health services among PWDs. Using the constant comparison approach, points of variation or convergence in the eligible studies were highlighted to derive key thematic and sub-thematic barriers. Two authors were involved in this process and where there was a disagreement, a third review author was consulted. | 7 |

Page 1 of 2

| **Section/topic** | **#** | **Checklist item** | **Reported on page #** |
| --- | --- | --- | --- |
| Risk of bias across studies | 15 | Quality appraisal of eligible studies assessed the study design, study aim, sampling procedures, role of confounding factors for potential bias and potential generalisability of findings using two widely used best practice quality appraisal tools: the CASP checklist^28^, and the quality assessment tool for surveys by the Center for Evidence-based Management (CEBMa)^29^. For all mixed-methods studies, the applicable quality appraisal tool was employed depending on the study design. These tools (CEBMa and CASP) are well established, scientifically rigorous and widely used and thus their external validity is not in doubt. The first and second authors of this paper led the quality assessment. Where discrepancies arose, a third reviewer acted as an arbiter. To optimize objectivity in the quality appraisal process, where reviewers are authors of eligible studies, they were not included in the quality assessment process. | 6 |
| Additional analyses | 16 | Not applicable |  |
| **RESULTS** | | |  |
| Study selection | 17 | In all, 1061 articles were retrieved from five electronic databases comprising Medline Complete = 472, CINAHL Complete = 372, PsycINFO = 220, Embase = 81, and Web of Science Core Collection = 23 (see Figure 1). An additional 39 articles were retrieved from other sources including reference list of eligible studies. Some 391duplicates were removed and the remaining screened from title and abstracts for relevance. Further, 658 studies were excluded after title and abstract screening leaving 51articles for which full text were obtained. At the end, 26 articles met the inclusion criteria and were retained for analysis. **Table 1** presents the characteristics of the eligible studies. | 7 |
| Study characteristics | 18 | Of the 26 eligible studies, 19 were qualitative in design^7,10,11,16,19,20,32–44^; four used a survey design^45–48^; and three used mixed methods design^49–51^. The 26 studies were reported from twelve African countries: Uganda (seven studies)^7,10,32,33,35,38,40^; Ghana (five studies)^11,40,50,52^; Zambia (five studies)^16,33,37,39,40^; Zimbabwe (two studies)^20,38^; South Africa (two studies)^37,45^; Kenya (one study)^31^; Namibia (one study)^43^; Senegal (one study)^19^; Nigeria (two studies)^46,49^; Rwanda (one study)^34^; Cameroon (two studies )^46,54^; and Ethiopia (three studies)^35,47,50^. | 7 |
| Risk of bias within studies | 19 | Ten out of the 19 eligible qualitative studies were rated as high quality^7,11,19,32,35–40^; two studies were medium to high quality^20,37^; while the remaining 7 studies were low to medium quality **(see Supplementary file 2)**. Most of the studies did not provide adequate information on how participants were recruited. Except one study^35^, none of the studies provided information on the relationship between researcher and participants (reflexivity). The quantitative studies^44–47^ had a medium to low quality rating **(****see supplementary file 3).** Lastly, the mixed methods studies^48–50^ were assessed as average or medium quality **(see supplementary file 4)**. | 8 |
| Results of individual studies | 20 | Overall, 33 specific barriers hindering access to SRH information and services among PWDs were identified. These barriers were further categorised into five levels: national, health system/institutional, individual, community and economic (see Table 2). Nine out of the 26 eligible studies identified three different set of barriers that PWDs face accessing SRH services at the national level^11,16,31–33,42,43^. These were unfriendly/lack of appropriate public transportation services^11,16,31,33,37,40,42^, and limited education opportunities for PWD on SRH issues^35,37^.  Eighteen different barriers were identified under the healthcare system/institutional level. These included lack of SRH information/resources at healthcare settings^20,32,42,43,48,50^; low staff capacity/numbers^38^; lack of adaptation of health information to suit PWDs^11,32,34,40,47^; lack of privacy and confidential services at the point of access^19,32–34,36,42,43,56^; lack of translators/sign language specialists^20,31,32,38,41,47^; frequent stock outs of essential SRH commodities^31,33^; lack of wheel chairs and mobility aids at the facility level^31,37^; unfriendly HIV/AIDS education materials^33^; longer waiting times^7,31–33,37^; and disability unfriendly physical healthcare infrastructure^7,31,32,35,37,40,42,43^. Other barriers included poor interpersonal relationships between PWDs and healthcare providers^37^; lack of knowledge or limited capacity of staff on PWDs SRH issues^7,11,16,32,33,40,42,48,50,56^; and insensitivity/negative attitudes of healthcare staff towards PWDs^7,11,19,20,31–33,35,39,40,42–44,47,49,56^. The rest comprised discrimination against PWDs by healthcare providers^19,20,31,32,34,36,42^; limited consultation time^48^; lack of access to HIV counseling and testing^45^; and limited availability of special outreach services for antenatal and postnatal care targeting persons with disabilities^37^.  Individual level barriers were the next most predominant. Sixteen studies reported seven different individual level barriers. These included gender inequalities^31–33,35,46^; negative socio-cultural/religious beliefs and practices^35,38,39^; refugee status^31^; low literacy rates among PWDs^48^; lack of knowledge/awareness on SRH issues^19,31,32,39,40,45,49^;communication barriers^11,19,33,34,36,39,44,49,50^; and lack of self-efficacy^16,38,48^.  Four different sets of community level barriers were identified in fourteen studies. These included negative public attitudes towards PWDs and their sexuality issues^7,11,16,19,20,31,33,37^; stigma and discrimination against disabled patients/clients^32,34,35,37,38^; sexual violence and abuse at the community level^19,31,33,34,39,45^; and lack of community or family support networks to enable PWDs access SRH services and information^11,19,20,32–35,37,39–41^.  Lastly, two main economic barriers emerged from review of eligible studies. These included unaffordability of SRH services and information^16,34,37,49^; and general financial and resource poverty which hinder PWDs’ access to SRH services and information^7,19,31,32,35,38,41^.  Overall, these barriers can be categorised into demand-side and supply-side barriers. For example, demand-side barriers relate PWDs’ lack of self-esteem, high level of illiteracy rates or lack of education, community and family level stigmatizatio which undermine access to SRH services, and lack of access to financial resources to access SRH services^11,20,36,53^. Supply-side barriers could include discrimination against PWDs. at health facilities by healthcare workers, disability unfriendly healthcare facilities, lack of disability-friendly wash rooms delivery/labour wards in healthcare facilities, and communication barriers between healthcare providers and PWDs^7,11,19,32,42^ . It is also important to note that whilst some of the barriers are peculiar to persons with specific types of disability, many other barriers are faced by the general population. For example, studies by Mphrah^51^ and Gichane^52^ show that persons with hearing difficulties (deaf) faced particular types of barriers including poor quality sign language interpretation services and inadequate knowledge about deaf people. These barriers specifically prevent them from accessing SRH services in healthcare facilities due to lack of effective interaction and communication systems. However, barriers such as lack of SRH information/resources at healthcare settings, low staff capacity/numbers, lack of privacy and confidential services at the point of access apply to the general public. | 8 and 9 |
| Synthesis of results | 21 | Not applicable |  |
| Risk of bias across studies | 22 | Ten out of the 19 eligible qualitative studies were rated as high quality^7,11,19,32,35–40^; two studies were medium to high quality^20,37^; while the remaining 7 studies were low to medium quality **(see Supplementary file 2)**. Most of the studies did not provide adequate information on how participants were recruited. Except one study^35^, none of the studies provided information on the relationship between researcher and participants (reflexivity). The quantitative studies^44–47^ had a medium to low quality rating **(see supplementary file 3).** Lastly, the mixed methods studies^48–50^ were assessed as average or medium quality **(see supplementary file 4)**. | 8 |
| Additional analysis | 23 | Not applicable |  |
| **DISCUSSION** | | |  |
| Summary of evidence | 24 | This paper appraised evidence on the barriers persons with disabilities face in accessing sexual and reproductive health information and services in sub-Saharan Africa. Five levels of barriers covering a total of 33 specific barriers were identified after pooling studies. The barriers identified comprised broader national level, healthcare system level, individual level, community level, and economic barriers. Many of the specific barriers identified however overlapped across studies, clinical settings and geographical contexts. Overall, these findings are largely consistent with previous related research on the barriers to sexual and reproductive health services in other contexts outside Africa^24,49–52^, and on access to general healthcare services^49,53,54^. For example, the present review identified lack of education and knowledge on sexual and reproductive health services and information, poor treatment of PWDs by healthcare workers, and disability unfriendly healthcare facilities and services. Studies in Nepal^5,56^, UK^57^, and India^55,58^ have reported similar findings. In a previous review covering low and middle-income countries, barriers to general healthcare services that persons with disability faced included lack of information, limited mobility, stigmatization, and negative and poor staff attitude^49^. This congruency underscores the fact that some of the barriers PWDs face in accessing sexual and reproductive health information and services may be global in nature and thus a well-concerted global response is needed. | 10 |
| Limitations | 25 | A number of limitations should be noted in this review. First, a number of barriers were identified as hindering access to sexual and reproductive health services among PWDs. However, this review did not indicate the extent to which such barriers interact or influence one another, and the ways in which such interactions determine access. Second, although a comprehensive search strategy was designed and conducted in five key biomedical and health sciences databases using broadly defined search terms, keywords and queries to identify and synthesize findings relevant to PWDs’ access to sexual and reproductive health services, only 26 articles were considered eligible for review. It is likely that some relevant articles were still missed due to language restriction. The search approach was restricted to only English language publications and it is plausible this resulted in the exclusion of eligible studies published in languages other than English. The search was also confined to only peer-reviewed journal articles, thus relevant editorials, theses, conference presentations which may have extended the depth of evidence on the topic were excluded. Third, this review adopted a multi-study design approach in enlisting eligible articles. Consequently, a meta-analysis was not permissible to assess the pooled effects of the barriers to accessing sexual reproductive health services. Another limitation of the study was the inability to establish confidence on the weight of the barriers to accessing SRH services by PWDs based on the overall frequencies. The GRADE tool may be considered in future reviews to establish the strength of the evidence presented in this review. Nevertheless, some strengths of the present review are notable. To the best of our knowledge, the present review presents the first attempt to comprehensively and systematically identify and synthesize both qualitative and quantitative studies on the barriers PWDs face in accessing sexual and reproductive health information and services in sub-Saharan Africa. A further strength of this review is that the search followed the PRISMA protocol, an internationally recognized best practice methodology in undertaking systematic reviews. | 12 |
| Conclusions | 26 | The findings of this review have implications for policy, practice and future research. From this review, it is clear that PWDs face myriad of both demand and supply side barriers to accessing sexual and reproductive health services and rights in sub-Saharan Africa. If the Sustainable Development Goals’ 3 objective of universal and/ or equitable access to skilled and comprehensive sexual, reproductive and maternal health services is to be attained in sub-Saharan Africa, urgent context-specific policy actions and disability-appropriate interventions are needed to address the barriers identified in this review. Barriers such as maltreatment of PWDs by healthcare professionals undermine the rights of PWDs to access sexual and reproductive health and rights. This requires policy and management attention to train healthcare providers on interpersonal communication skills and relationships. This could enable healthcare providers deliver healthcare services with high level of sensitivity and fairness. Similarly, limited availability of access ramps posed a great deterrent to access to services among PWDs.^11,32,38^. This requires management of health facilities to ensure adequate provision of access ramps to facilitate better access for persons with physical disabilities. Also, some healthcare workers lacked the requisite professional skills to deal with PWDs. Therefore, we advocate further and regular training of healthcare workers on how to provide sexual and reproductive healthcare information and services to PWDs in a respectful and non-judgmental manner. Specific courses on providing care to disabled persons should be incorporated into the curricular of health training institutions. Training manuals on this topic should also be made available, with such trainings segregated according to the types of disability, their culture and unique healthcare needs. This will ensure such trainings are context-specific and seek to identify and address the needs of specific disability groups.  Another issue relates to the lack of support from communities and families, which is fueled by misperceptions about PWDs and their sexuality. These misperceptions and beliefs are borne out of poor understanding of disability as well as lack of awareness about the sexuality and sex life of PWDs. It is therefore important that public educational interventions are designed and implemented to demystify such prevailing beliefs and practices, improve public understanding of the sexual and reproductive health needs of PWDs and ways the public could support PWDs to fully enjoy their fundamental human rights in relation to safe and satisfying sexual life. In a similar vein, PWDs should be educated more on issues relating to their sexual rights and access to sexual and reproductive health services. This is important to overcome lack of awareness on sexual and reproductive health issues and lack of self-efficacy among PWDs. Indeed, insights from this review could be used to develop an evidence-based implementation strategy on how to address access barriers at the various levels: national level, institutional or health system context, economic context, individual PWDs, community and family level contexts. This could, for example, include developing training guidelines and tools for instruction at the various health training institutions for healthcare providers.  Finally, although different types of disabilities exist, the review showed that there is relatively more scholarly attention on particular types of disabilities compared to others. The studies reviewed reported more on hearing/speech impairments, visual impairment and physical disability and less on other types of disability such as mental or intellectual disability. Further studies are thus required to bring to light the barriers faced by people with these types of disabilities. The international literature on PWDs suggests that females face more barriers in general compared to their male counterparts. This was however not clearly articulated in this review. We suggest that future research delves further into the gender-based barriers PWDs face in accessing sexual and reproductive health services. This will ensure that existing health programmes and interventions are sensitive to, and addresses, the unique needs of both females and males.  In conclusion, persons with disabilities face a myriad of demand and supply side barriers to accessing sexual and reproductive healthcare in Sub-Saharan Africa. Multilevel interventions are urgently needed to address these barriers | 10-12 |
| **FUNDING** | | |  |
| Funding | 27 | The author(s) received no specific funding for this work. | 13 |

*From:*  Moher D, Liberati A, Tetzlaff J, Altman DG, The PRISMA Group (2009). Preferred Reporting Items for Systematic Reviews and Meta-Analyses: The PRISMA Statement. PLoS Med 6(7): e1000097. doi:10.1371/journal.pmed1000097

For more information, visit: **www.prisma-statement.org**.

Page 2 of 2
