# Supplementary material for: Barriers facing persons with disability in accessing sexual and reproductive health services in sub-Saharan Africa: A systematic review
Source: PLoS One. 2020 Oct 12;15(10):e0238585. doi: 10.1371/journal.pone.0238585 (PMC7549766; doi:10.1371/journal.pone.0238585)
Supplement: S1 File — (DOCX) [file pone.0238585.s002.docx]

**S1 File: Medline Search Strategy**

| Search | Query |
| --- | --- |
| 1 | (Disability) OR (Disabilities) OR (Impairment) OR (Hearing Impairment) OR (Persons with Disability) OR (Physical and Sensory Impairment) OR (Developmental Disability) OR (Developmental and Intellectual Disability) OR (Deaf) OR (Visual) OR (Blind ) OR (Intellectual Disability) OR (Psychosocial Disability) OR (Albinism) OR (Physical Disability) OR (Mental Disability) |
| 2 | (Reproductive Health) OR (Sexual Health) OR (Sexual Education) OR (Sexuality Education) OR (Sexual Health Information) OR (Family Planning Services) OR (Antenatal Care) OR (In-facility Delivery) OR (Antenatal Care) OR (Postnatal Care) OR (Modern Contraception) OR (Abortion) OR (Contraceptives) OR (Condom) OR (Pregnant Women) OR (Health Services) OR (Emergency Obstetric Services) OR (Obstetric Complications) OR (Maternal Health) or (Child Health) OR (Skilled Birth Attendance) OR (Sexuality) OR (Adolescent) OR (Sexuality and Disability) OR (Motherhood) OR (Reproductive Health Services) OR (Birth Control) OR (Pills) OR (Intrauterine Device) OR (IUD) OR (Emergency Contraception) OR (Sterilization) OR (Spermicide) OR (Diaphragm) OR ( Cervical Cap) OR (Contraceptive Implant) |
| 3 | (Barriers) OR (Challenges) OR (Experiences) OR (Impediments) OR (Access) OR (Inhibiters) OR (Uptake) OR (Utilization) OR (Compliance) OR (Adherence) |
| 4 | (Africa) OR (Africa South of the Sahara) OR (sub-Saharan Africa ) OR (Central Africa) OR (Southern Africa) OR (Eastern Africa ) OR (Western Sahara) OR (East Africa ) OR (Central African Republic ) OR (West Africa ) OR (Cameroon) OR (Chad) OR (Congo) OR (Democratic Republic of Congo) OR (Congo, Demographic Republic) OR (Congo, Republic) OR (Equatorial Guinea) OR (Gabon) OR (Burundi) OR (Djibouti) OR (Eritrea ) OR (Ethiopia ) OR (Kenya) OR (Rwanda) OR (Somalia) OR (Sudan) OR (Tanzania) OR (Uganda) OR (Angola) OR (Botswana) OR (Lesotho) OR (Malawi) OR (Mozambique) OR (Namibia) OR (Swaziland) OR (Zambia) OR (Zimbabwe) OR (Benin) OR (Burkina Faso) OR (Cape Verde) OR (Cote D'ivoire) OR (Gambia) OR (Gambia, The) OR (Ghana) OR (Guinea) OR (Guinea-Bissau) OR (Liberia) OR (Mali) OR (Mauritania) OR (Niger) OR (Nigeria) OR (Senegal) OR (Sierra Leone) OR (Togo) OR (South Sudan) OR (Madagascar) OR (Comoros) OR (Mauritius) OR (Sao Tome and Principe) OR (Seychelles) OR (South Africa) |
| 5 | 1 AND 2 AND 3 |
| 6 | 4 AND 5 |
| Limiters | 2001-2020  Full Text  English Language  Academic/Peer reviewed Journals  Humans |
