# Supplementary material for: Barriers facing persons with disability in accessing sexual and reproductive health services in sub-Saharan Africa: A systematic review
Source: PLoS One. 2020 Oct 12;15(10):e0238585. doi: 10.1371/journal.pone.0238585 (PMC7549766; doi:10.1371/journal.pone.0238585)
Supplement: S2 File — (DOCX) [file pone.0238585.s003.docx]

**S2 File:** Quality assessment results of qualitative included studies (CASP)

| **Quality assessment questions** | Burke et al, 2017 | Mavuso & Maharaj, 2015 | Bremer et al, 2009 | Rugoho & Maphosa, 2015 | Ahumuza et al, 2014 | Ganle et al, 2016 | Peta et al, 2017 | Yousafzai et al, 2009 | Tun et al, 2015 | Mprah, 2013 | Tanabe et al, 2015 | Mulumba et al, 2014 | Smith et al, 2004 | Rooy & Mufune, 2014 | Schenk et al 2020 | Apolot et al, 2019 | Parsons et al, 2015 | Nixon et al, 2014 | Tefera et al, 2017 |
| --- | --- | --- | --- | --- | --- | --- | --- | --- | --- | --- | --- | --- | --- | --- | --- | --- | --- | --- | --- |
| Was there a clear statement of the aims of the research? | Yes | Yes | Yes | Yes | Yes | Yes | Yes | Yes | Yes | Yes | Yes | Yes | Yes | Yes | Yes | Yes | Yes | Yes | Yes |
| Is a qualitative methodology appropriate? | Yes | Yes | Yes | Yes | Yes | Yes | Yes | Yes | Yes | Yes | Yes | Yes | Yes | Yes | Yes | Yes | Yes | Yes | Yes |
| Was the research design appropriate to address the aims of the research? | Yes | Yes | Yes | Yes | Yes | Yes | Yes | Yes | Yes | Yes | Can’t tell | Yes | Yes | Yes | Yes | Yes | Yes | Yes | Yes |
| Was the recruitment strategy appropriate to the aims of the research? | Yes | Yes | Yes | No | Yes | Yes | Can’t tell | Can’t tell | Yes | Yes | Yes | Yes | Can’t tell | Can’t tell | Yes | Yes | Yes | Yes | Yes |
| Was the data collected in a way that addressed the research issue? | Yes | Yes | Yes | Yes | Yes | Yes | Yes | Yes | Yes | Yes | Can’t tell | Yes | Yes | Can’t tell | Yes | Yes | Yes | Yes | Yes |
| Has the relationship between researcher and participants been considered? | Can’t tell | No | Yes | No | No | No | No | No | No | No | No | No | No | No | No | No | No | No | No |
| Have ethical issues been taken into consideration? | Yes | Yes | Yes | Yes | Yes | Yes | Yes | No | Yes | Yes | Yes | Yes | No | No | Yes | Yes | Yes | Yes | Can’t tell |
| Was the data analysis sufficiently rigorous? | Yes | Can’t tell | No | Can’t tell | Yes | Yes | Yes | No | Yes | No | No | Yes | Yes | No | Yes | Yes | Yes | Yes | Yes |
| Is there a clear statement of findings? | Yes | Yes | Yes | Yes | Yes | Yes | Yes | Yes | Yes | Yes | Yes | Yes | Yes | Yes | Yes | Yes | Yes | Yes | Yes |
| How valuable is the research? | Yes | Yes | Yes | Yes | Yes | Yes | Yes | Yes | Yes | Yes | Yes | Yes | Yes | Yes | Yes | Yes | Yes | Yes | Yes |
