# Supplementary material for: Barriers facing persons with disability in accessing sexual and reproductive health services in sub-Saharan Africa: A systematic review
Source: PLoS One. 2020 Oct 12;15(10):e0238585. doi: 10.1371/journal.pone.0238585 (PMC7549766; doi:10.1371/journal.pone.0238585)
Supplement: S3 File — (DOCX) [file pone.0238585.s004.docx]

**S3 File:** Quality assessment results of quantitative included studies (CEBM)

| **Quality assessment questions** | Gichane et al, 2017 | Beyene et al, 2019 | DeBeaudrap et al, 2019 | Oladunni, 2012 |
| --- | --- | --- | --- | --- |
| Did the study address a clearly focused question/issue? | Yes | Yes | Yes | Yes |
| Is the research method (study design) appropriate for answering the research question? | Yes | Yes | Yes | Can’t tell |
| Is the method of selection of the subjects (employees, teams, divisions, organizations) clearly described? | Yes | Can’t tell | Yes | No |
| Could the way the sample was obtained introduce (selection) bias? | Can’t tell | Yes | Can’t tell | No |
| Was the sample of subjects representative with regard to the population to which the findings will be referred?? | Can’t tell | No | Yes | Yes |
| Was the sample size based on pre-study considerations of statistical power? | No | No | Can’t tell | Yes |
| Was a satisfactory response rate achieved? | Can’t tell | Yes | Yes | Yes |
| Are the measurements (questionnaires) likely to be valid and reliable? | Yes | Can’t tell | Yes | Can’t tell |
| Was the statistical significance assessed? | No | Yes | Yes | No |
| Are confidence intervals given for the main results? | No | Yes | Yes | No |
| Could there be confounding factors that haven’t been accounted for? | Yes | Can’t tell | No | Yes |
| Can the results be applied to your organization? | Yes | Yes | Yes | Yes |
