# Supplementary material for: Barriers facing persons with disability in accessing sexual and reproductive health services in sub-Saharan Africa: A systematic review
Source: PLoS One. 2020 Oct 12;15(10):e0238585. doi: 10.1371/journal.pone.0238585 (PMC7549766; doi:10.1371/journal.pone.0238585)
Supplement: S4 File — (DOCX) [file pone.0238585.s005.docx]

**S4 File:** Quality assessment results of mixed methods design using both CASP & CEBM tools.

| **Quality assessment questions for qualitative methods sections** | **Mprah et al, 2017** | **Oladunni, 2012** | **Yimer & Modiba, 2019** |
| --- | --- | --- | --- |
| Was there a clear statement of the aims of the research? | Yes | Yes | Yes |
| Is a qualitative methodology appropriate? | Yes | Yes | Yes |
| Was the research design appropriate to address the aims of the research? | Yes | Yes | Yes |
| Was the recruitment strategy appropriate to the aims of the research? | Yes | Can’t tell | Can’t tell |
| Was the data collected in a way that addressed the research issue? | No | Yes | Yes |
| Has the relationship between researcher and participants been considered? | No | No | No |
| Have ethical issues been taken into consideration? | Yes | No | Yes |
| Was the data analysis sufficiently rigorous? | Yes | No | Yes |
| Is there a clear statement of findings? | Yes | Yes | Yes |
| How valuable is the research? | Yes | Yes | Yes |
| **Quality assessment questions for quantitative (survey) methods sections** | Yes | Yes |  |
| Did the study address a clearly focused question/issue? | Yes | Yes | Yes |
| Is the research method (study design) appropriate for answering the research question? | Yes | Yes | Yes |
| Is the method of selection of the subjects (employees, teams, divisions, organizations) clearly described? | Yes | No | Can’t tell |
| Could the way the sample was obtained introduce (selection) bias? | No | Yes | No |
| Was the sample of subjects representative with regard to the population to which the findings will be referred? | Can’t tell | Can’t tell | No |
| Was the sample size based on pre-study considerations of statistical power? | No | No | No |
| Was a satisfactory response rate achieved? | Can’t tell | Can’t tell | Yes |
| Are the measurements (questionnaires) likely to be valid and reliable? | Can’t tell | Can’t tell | Yes |
| Was the statistical significance assessed? | No | No | Yes |
| Are confidence intervals given for the main results? | No | No | Yes |
| Could there be confounding factors that haven’t been accounted for? | Yes | Yes | No |
| Can the results be applied to your organization? | Yes | Yes | Yes |
