# Supplementary material for: Barriers facing persons with disability in accessing sexual and reproductive health services in sub-Saharan Africa: A systematic review
Source: PLoS One. 2020 Oct 12;15(10):e0238585. doi: 10.1371/journal.pone.0238585 (PMC7549766; doi:10.1371/journal.pone.0238585)
Supplement: Attachment — Submitted filename: Response to Reviewer.docx [file pone.0238585.s006.docx]

**Journal:**	PLOS ONE

**Manuscript ID:**	PONE-D-20-07319

**Manuscript Title:**	Barriers facing persons with disability in accessing sexual and reproductive health services in Sub-Saharan Africa: a systematic review

**Authors:**	John Kuumuori Ganle, Leonard Baatiema, Reginald Quansah & Anthony Danso-Appiah

**REVISION NOTES TO MANUSCRIPT**

| **S/N** | **Reviewers’ Comments** | **Response** |
| --- | --- | --- |
| **Reviewer 1** | Authors have stated the rationale for this review vividly and it is within the context of what is already known. However, the statement that the evidence has not been systematically documented in Sub-Saharan Africa, to me appears weak and the statement has been emphasized every section of the text. it should be reworked on based on factors to be studied and should appear only in the introduction and abstract section. What is reason for consolidating the papers? The fact that the papers have not been systematically documented does not translate to a knowledge gap. Can you come up with an appropriate reason for your review? | We are encouraged by the reviewer’s comment that the rationale for our review has been vividly stated within the context of what is already known. We have revised the manuscript to provide addition justification/reason for this review. Our revision now reads: ***‘‘While these studies provide useful evidence on the barriers to accessing sexual and reproductive health services among PWDs in the individual contexts within which they have been conducted, no systematic review has been conducted to pull together these pieces of research evidence for us to understand the nature, magnitude and extent of these barriers in different settings in sub-Saharan Africa. Moreover, we do not yet have a good understanding of the strength/quality of the evidence that exist on the barriers persons with disabilities face when accessing sexual and reproductive health services in sub-Saharan Africa. This evidence gap could potentially undermine sub-regional planning and efforts to develop more inclusive sexual and reproductive healthcare policies and programmes that have the potential to propel progress towards the Sustainable Development Goals’ 3 objective of universal and/ or equitable access to skilled and comprehensive sexual, reproductive and maternal health services’’.* PLEASE, SEE PAGES 3/4, LINES 111-132 OF THE REVISED MANUSCRIPT.** |
|  | It would help readers if you provided a nice and clear definition of 'disabilities' the Introduction and also, a sentence or two outlining its impact globally, i.e. why/how access to sexual and reproductive health services is a problem/important for this particular group? | On page 3, lines 82-84, we have provided a definition of disability based on the Wealth Organization World’s 2011 World Report on Disability. While there may be other discipline or theory specific ways of defining disability, we think the defintion provided in the World Report on Disability is the most commonly used in literature.  Also, we have provided more information the global impact of disability and why/how access to sexual and reproductive health services is a problem/important. Please, see lines 86-90 of the revised manuscript. |
|  | Methods: It is no longer recommended that searching and screening be restricted to English-only as this can introduce language bias. Your statement of eligibility “with only English Language restriction” is already present bias. Majority of abstracts will provide an English translation for title and abstract screening; and there are online translation programs (e.g. Google Translate) that will normally provide an understandable English translation of full text reports. So your criteria for language should read 'must be in English or able to translate into understandable English' | We thank the Reviewer for raising this very critical issue in relation to restricting review searches to only English language publications. We acknowledge that our decision to restrict the search on only English language publications risks introducing language bias. We were however constrained by time and resources to engage translation services for articles published in languages other than English. However, this has been acknowledged as a study limitation. |
|  | Search strategy, should be detailed, explicable and with relevant key words/search terms. I tried to google and the feedback with information retrieved were overwhelming. Come up with a sub-heading detailing the inclusion and exclusion criteria used | We have provided a comprehensive list of key words and search terms in supplementary file 1. We believe the list of key words presented there captures almost all the relevant keywords and Medical search terms in relation to the study topic. Regarding the Reviewer’s suggestion to incorporate a sub-heading for inclusion and exclusion criteria, this has already been outlined in page 4 and part of page 5. |
|  | It is nice to see that you tried to frame your research question in PICO format however, without the specific question to help you address those concern, it would be like speculation. Please sentence of a sentence of a research question that begin…”what is the evidence that….. | We are also encouraged by the reviewer’s comments that we tried to frame our research questions in PICO format. We have revised the manuscript to include the specific question/s that we sought to answer. Our revision now readS: *“To address gap, this systematic review aimed to answer the following inter-related research questions: what is the evidence that PWDs face barriers in accessing sexual and reproductive health (SRH) information and services in sub-Saharan Africa; and what specific barriers do PWDs face in accessing sexual and reproductive health (SRH) information and services in sub-Saharan Africa?’’* **PLEASE, SEE PAGE 4, LINES 132-137 OF THE REVISED MANUSCRIPT.**  the |
|  | Study selection: You do not need to mention the initial of the authors in the text (same with data extraction). There is a section of author role dedicated by the journal for this. However, you need to explain chronological event involved in the study selection and that disagreement between two reviewers to be resolved by a third reviewer, if you invite two or more reviewers as in between a further disagreement may ensue and the circus continues…. | The initials of the reviewers have now been removed from the text. The search process, study selection, data abstraction, quality appraisal and analysis have been reported chronologically. Thank you |
|  | How would you manage issues of missing data? Please explain | As our review did not involve meta-analysis and quantification or pooling of effects, we did not have much challenge with missing data. However, where some eligible studies had missing data or presented insufficient published data, one reviewer contacted the study’s corresponding author to clarify the missing data and retrieve same where the data was able. We have revised our manuscript to clarify this. **Please, see page 6, lines 216-218 of the revised manuscript.** |
|  | Now that improvement in the assessment of evidence is currently in the works. It would be important to consider use of GRADE tool for quality ratings. I observed that you focused on both qualitative and quantitative data, and stated that Most of the studies did not provide adequate information on how participants were recruited. In addition, that the quantitative studies had a medium to low quality rating. As such methods may differs in terms of quality of evidence, thus GRADE should take care of risk of bias across studies, publication bias among others. Note: I was not able to access supplementary file 3 and 4 for verification. | We agree with the Reviewer about employing the GRADE instrument for quality ratings. Evidence about using the GRADE tool to measure the confidence of reviews findings or overall evidence is increasingly used but still remains a field under development. We thank the Reviewer for recommending the GRADE tool and this will be considered in future reviews. This has been acknowledged as a study limitation (page 11). |
| **Reviewer 2** | The authors should be consistent with the use of sub-Saharan Africa | We have revised and now maintained the spelling of sub-Saharan African throughout the text. Thank you |
|  | Page 3 Line 71 and the entire manuscript, Please check the referencing style of PLOS ONE and reformat them accordingly | The references have been re-checked and revisions accordingly made to ensure consistency and alignment with PLOS ONE recommendation. |
|  | Page 3 Line 75 and line 124 authors have defined their abbreviation, PWDs (Persons with Disability). Please start the sentences with PWDs instead of Persons with Disability | The revision has been made as recommended. |
|  | The authors might wish to consider a recent study “Kumi-Kyereme, A., Seidu, A. A., & Darteh, E. K. M (2020). Factors Contributing to Challenges in Accessing Sexual and Reproductive Health Services Among Young People with Disabilities in Ghana. Global Social Welfare, 1-10” at their literature review section or background  . <https://www.researchgate.net/publication/341578212_Factors_Contributing_to_Challenges_in_Accessing_Sexual_and_Reproductive_Health_Services_Among_Young_People_with_Disabilities_in_Ghana> | We welcome this recommended article as we found it very useful. Insights from this study has since been incorporated in this revised version as seen in lines 98 and 319. |
|  | Page 4 Line 127 what informed the authors to use age 15 as the starting point? | We have revised the manuscript to explain why we started with 15years and above. Our revision reads: *“While both age of menarche among girls and sexual debut among boys and girls have declined in recent years, most international policy and research on sexual and reproductive health often focus on age 15 onwards as the starting point of sexual activity and reproduction^11,16,17^. Our focus on 15years and above was informed therefore informed by this international policy and research literature on sexual and reproductive health’’.* PLEASE, SEE PAGES 5, LINES 172-176 OF THE REVISED MANUSCRIPT |
|  | Page 6 Line 174, kindly replace “reviews” with “reviewers” | This has been revised as suggested. Thank you. |
|  | Page 6 Line 194 specify these two authors | To ensure consistency and avoidance of repetitions, the authors have removed the initials/identities of the reviewers in the main text given that full details about their roles are outlined at the end of the manuscript. |
|  | Page 7 Line 210 to 216 and line 233-237 please take a critical look at the use of comma (,) | We have done this. Where appropriate, we have replaced the commas (,) with semi-colons (;). |
|  | Page 9 Line 274-275 kindly replace developing country with low and middle-income countries | We have now replaced developing country with low and middle-income countries |
|  | Page 9 between line 279 and 280 consider creating a sub-section “Strengths and Limitations” | We have now revised the manuscript to include a section on study limitations. |
|  | Similarly, Kindly consider creating a subsection “implications for policy, practice and future research” between line 297 and 298. | We thank the Reviewer for this suggestion. However, we are unable to accommodate this suggestion as this would adversely affect the coherence and logical presentation of our presentation. |
|  | Although the authors have presented/fused their conclusions at the “implications for policy, practice and future research” section, it will be appropriate to have a separate heading/section for the conclusion they drew from their review | We now have a separate section for the study conclusion. |
| **Reviewer 3** | Since we just in the middle of 2020, what month was the search conducted? how many 2020 paper were included? perhaps capping the study period to 2019 will be fine, otherwise, state the month in 2020 the search ended. | Please we have indicated on page 5 under search strategy the month in 2020 when the search was undertaken. Thank you. |
|  | Since this study is about SSA, I wonder why AJOL, where many African Journals are represented was not searched. If there a justification for this? | We agree with the Reviewer about the recommendation to have included AJOL as part of our searched databases given its African focus. However, we believe the databases included enabled us to retrieve all relevant eligible articles included in the study and thus the possibility of excluding other eligible studies is very unlikely. We will endeavor to include AJOL in future searches. |
|  | Authors did not discuss the limitations of this study. The study only came from 12 of over 40 SSA countries. To what extent is the study representative of the SSA? | Please see page 11 of the manuscript for an elaborated section on study limitations and strengths. Based on the search strategy, the use of both qualitative and quantitative studies for this review, we believe the findings from this review has the potential to be representative of SSA taking into account the acknowledged study limitations. Thank you |
|  | Did authors reviewed international frameworks and SSA countries policies on people with disability and what was found. Policies on people with disabilities in SSA will help provide more context to the findings of the study. | This was outside the scope of the present review although we agree with the reviewer this would have generated very insightful evidence to inform policies seeking to improve access to SRH services by PWDs. |
| **Reviewer 4** | The manuscript addresses a topical issue given the vulnerability of PWD and global recognition by the International Convention on the Rights of Persons with Disability (CRPD) adopted in 2006 by the United Nations; as well as the SDG slogan of *Leaving no one behind*. | We are encouraged by the review’s comments that our manuscript addresses a topical issue in the context of both the International Convention on the Rights of Persons with Disability (CRPD) and the SDG slogan of *Leaving no one behind*. We have revised the manuscript to incorporate the very useful suggestions the reviewer has made. |
|  | The results are basically summarized on pages 7-8, under two sub-headings: *characteristics of the studies included* and ***Synthesis of barriers to SRH services among PWDs.*** Identified barriers have been categorized into five levels (namely: national, health system/institutional, individual, community and economic). The synthesis is presented in the form of a quantitative summary. For instance: *Nine out of the 26 eligible studies identified three different sets of barriers that PWDs face accessing SRH services at the national level. These were unfriendly legal frameworks and policies...*. It will be helpful to provide more details here including a clear indication as to what these *unfriendly legal frameworks and policies* consist of. Also, since this is the main focus of the manuscript, it would be more informative to provide a synthesis of the key barriers, especially highlighting the most common and the specific ones. It should be noted that some barriers may depend on type and even level /extent of disability.  Moreover, the barriers listed actually include some that may not necessarily be specific to PWD but are rather general in nature (lack of SRH information/resources at the various healthcare settings, low staff capacity/numbers, lack of privacy and confidential services at the point of access). It is not clear why these considered as barriers are faced by PWDs and not the entire population! | We thank the Reviewer for these relevant suggestions. We have now revised the section on synthesis of barriers to SRH to clarify the categories of barriers faced by PWDs, highlighting barriers specific to particular types of disability. Our revisions have also provided further distinction on the barriers for both the demand and supply-side level barriers. please refer to page 9 (lines 262-281) |
|  | It is also interesting to have the concluding statements suggesting that PWD faced myriad of both ***demand and supply side barriers*** to accessing sexual and reproductive health services and rights in Sub-Saharan Africa. Whereas, there was no clear distinction made as to the demand and supply side barriers identified. It is therefore important to further categorize the barriers into these two broad groups. | We have now revised the manuscript to report on the demand-side and supply-side categories of barriers faced by PWDs. |
|  | Figure 1 and Table 1, which present the main results of the study seem to have some inconsistencies. The total number of studies included as per my estimation is 25 (instead of the 26 reported). Furthermore, Table 1 provides the composition of studies participants (gender, age, disability status, etc.) for some but not all studies listed | We have checked the number of studies included and the total **is 26 as can be seen in table 1**. Where applicable, information on the composition of study participants were reported. |
|  | Lines 152-174 sub-titled: Study selection and data extraction is basically highlighting what is already captured under the authors' contribution. | We have now revised lines 152-174 to avoid repetitions. Thank you. |
|  | Lines 208-209 states that: *At the end, 26 articles involving* ***1499 participants*** *met the inclusion criteria and were retained for analysis.* The significance of this number of participants here or to the outcome of the current analysis is not clear! Furthermore, it is uncertain how this number was arrived at from Table 1 (referenced) since some studies listed therein do not provide the number of participants | We agree with the Reviewer on this point. We have revised lines 208-209 by deleting the information about the number of participants accordingly. |
